# Books and Authors

**Published:** 1909-07

**Authors:** 


### BOOKS AND AUTHORS.

---

**Elementary Practical Treatise on Diseases of the Larynx and Pharynx** by **Dr. E. J. Moure,** Surgeon in charge of the Nose, Ear and Throat Department of the Faculty of Medicine, Bordeaux. Translated and adapted by **J. Malcolm Farquharson,** M.B., F.R.C.R., Edin., Lecturer on Diseases of Nose, Ear and Throat in the School of Medicine of the Royal College, Edinburgh, etc., with 210 illustrations. New York: Rebman Company. 1909.

This author has departed from the usual lines in constructing this work. It is limited in its observations to a field below the nasal cavity, and deals with the pharynx and larynx alone, or strictly speaking, together, for the first time in an elaborate treatise. The book is an enlargement of the author's lectures published in 1890, adding the pathology of the tonsils, soft palate, pharynx, and the lingual tonsil. Bacteriology has developed into a large field since the author's lectures were published, and this fact has assisted largely to make the pathology of the pharynx better understood. Therapeutics, too, occupy an increased attention commensurate with other advances.

A number of inflammatory conditions are set forth that do not find place even in the more recent manuals and treatises, such as erythematous angina, erythema polymorphia, pemphigus, and acute ulcerative lacunar tonsillitis, the latter here being differentiated from ulcerative membranous tonsillitis, with which it is often confounded. The author makes a classification of his own as regards tonsillar abscesses, basing it upon the origin of these suppurative processes and upon their history. He evolves a special plan of treatment based on these observations. He omits a description of the anginæ symptomatic of rheumatism, scarlet fever and other exanthemata, which are best treated in works on general medicine.

The second part, devoted to diseases of the larynx, is entirely modern. For example, the author describes Kirstein's direct method of tracheoscopy, and Killian's tracheo-bronchoscopy. Pathology again receives particular consideration. By and large it is a work that has not its superior on these topics. It contains much material of special assistance to the general practitioner, while, at the same time, the specialist cannot afford to go without it. The illustrations are excellent, many of which are works of art. The publishers have made it into a handsome book.

**Conservative Gynecology and Electro Therapeutics:** A Practical Treatise on the Diseases of Women and Their Treatment by Electricity by **G. Betton Massey,** M.D., Attending Surgeon to the American Oncologic Hospital, Philadelphia; Fellow and Ex-President of the American Electro-Therapeutic Association; Member of the American Medical Association, etc. Sixth edition, thoroughly revised. Royal octavo, 462 pages. Illustrated with twelve (12) original, full-page, chromo-lithographic plates and fifteen (15) full-page half-tone plates of photographs taken from nature, and numerous engravings in the text. Bound in extra cloth. F. A. Davis Company, publishers, 1914-16 Cherry street, Philadelphia, Pa. (Price, $4.00 net.)

From the viewpoint of the electrotherapist it would be difficult to make a better book for the purpose intended by this anthor.

Moreover, it is acknowledged as a safe guide on electrotherapeutics as applied to gynecology. The author is an industrious student of the subject, and presents it in an attractive form. The illustrations are superb, especially the half-tones of clinical demonstrations. This being the sixth edition, a careful revision has been made, and a new chapter added on the principles of electro-chemical surgery in the removal of new growths, the treatment of tubercular adenitis by zinc-mercury ions, and the use of high-frequency, high potential currents in gynecology. The book is now at its best.

